# Impact of marital status during diagnosis on cancer-caused specific survival in acute myeloid leukemia patients: a case-control and population-based study

**DOI:** 10.18632/oncotarget.16989

**Published:** 2017-04-09

**Authors:** Zhuojun Zheng, Yuandong Zhu, Xiaodong Li, Wenwei Hu, Jingting Jiang

**Affiliations:** ^1^ Department of Hematology, The Third Affiliated Hospital of Soochow University, Changzhou, China; ^2^ Department of Tumor Biological Treatment, The Third Affiliated Hospital of Soochow University, Changzhou, China; ^3^ Cancer Immunotherapy Engineering Research Center of Jiangsu Province, Changzhou, China; ^4^ Institute of Cell Therapy Soochow University, Changzhou, China; ^5^ Department of Oncology, The Third Affiliated Hospital of Soochow University, Changzhou, China

**Keywords:** acute myeloid leukemia, marital status, SEER, survival analysis, subgroup analysis

## Abstract

**Objective:**

This study investigated the impact of marital status on cancer-caused specific mortality among acute myeloid leukemia (AML) patients in the United States.

**Methods:**

We used the Surveillance, Epidemiology and End Results program to identify 50,825 patients who had their clinical and follow-up information available and were diagnosed for AML between the years 1988 and 2015. The univariate and multivariable Cox regression models were used to analyze the patient data, and to minimize the group differences due to covariates between groups, a 1:1 propensity score matching was used in subsequent subgroup analysis.

**Results:**

Our study demonstrated that married patients were less likely to die due to AML after adjusting for demographic and clinicopathological variables, than patients with variable unmarried status. Further analysis indicated that widowed, divorced and never married status correlated with poor cancer-cause specific survival than being married in almost all subgroups after being adjusted for the aforementioned variables (*P*<0.05). However, the difference between married and separated was not apparent. Moreover, similar survival analysis results were also observed in the 1:1 matched subgroups of marital status, but they displayed varied prognostic factors between them. The association of survival benefit with marriage in AML was consistent with the published survival benefit of conventional therapeutic approaches.

**Conclusion:**

Overall, our study concluded that unmarried AML patients were at greater risk of cancer-specific mortality than married, and thus indicated that physicians should focus on health care strategies that target social support, in order to reduce the cancer-specific mortality in unmarried patients.

## INTRODUCTION

Among the hematological malignancies, humans usually display high frequency of acute leukemia. In adults, acute myeloid leukemia (AML) is more common than acute lymphoblastic leukemia, and its incidence gradually increases with age and rate is about 16.2 cases per 100,000 individuals with age over 65 year. The underlying mechanism of AML is still unknown, and the only significant therapeutic advantage achieved in the last decade has been the long-term cure of patients with acute promyelocytic leukemia (APL) while invasion is absence in advance of non-APL. Currently, the 5-year overall survival rate is between 0 to 40%, and complete remission rate is ≥80% in younger patients, while it is disappointingly very low in older patients [1].

The social support among the many factors actually exerts a significant effect on the clinical outcome, especially in patients with malignant diseases [2, 3]. It is well known that marital status has been the most important social support, which also associates with a variety of other important social factors [4]. Married patients generally show better recovery from a single malignancy, as they seem to receive more social support, including practical support and financial resources. Some studies have demonstrated that marital status is an independent prognostic factor in multiple cancers [5-8], and the survival benefits due to marriage has been greater than the published survival benefits from chemotherapy in several cancers [9-12]. In addition, marital status linkage with delayed diagnosis, lack of treatment and social support, leads to poor survival [5, 13, 14]. There has been conflicting studies about the effect of marriage on acute leukemia. For instance, the study by Borate *et. al.* showed that single (never married) and divorce status were poor prognostic factors for survival in younger AML patients [15]. However, on the contrary, the study by Fintel *et. al.* [16] reported that marriage did not have any influence on the survival outcome in adolescent and young adults with AML, thereby suggesting that social issues like marital status were less important than disease-specific therapies. Thus, we believe that a better understanding of the impact of marital status on AML would lead to better understanding of the importance of social mechanisms in the management of this malignancy and can help to establish a more holistic approach to improve patient outcomes. Hence, we in this study have tried to explore the underlying mechanisms of the correlation between marital status and the survival of younger and elderly AML patients.

## MATERIALS AND METHODS

### Data source

This study used Surveillance, Epidemiology, and End Results (SEER) database released in November 2015 as a data source. It included data from 18 population-based registries from 1973 to 2013 and covers approximately 30% of the US population. The SEER program registries routinely collected data on patient demographics, primary tumor site, tumor morphology and stage at diagnosis, first course of treatment, and follow-up for vital status. The mortality data reported by SEER were updated annually by the National Center for Health Statistics [17]. The National Cancer Institute’s SEER*Stat software (Surveillance Research Program, National Cancer Institute SEER*Stat software, www.seer.cancer.gov/seerstat) (Version 8.3.2) was used to collect all the necessary information.

### Inclusion criteria

To identify appropriate patients for this study, the following inclusion criteria was used: a) Patients should be diagnosed with AML (International Classification of Diseases for Oncology, Third Edition [ICD-O-3], codes 9840/3, 9861/3, 9865/3, 9866/3, 9867/3, 9869/3, 9871/3, 9872/3, 9873/3, 9874/3, 9895/3, 9896/3, 9897/3, 9898/3, 9910/3, 9911/3, 9920/3), between the year 1988 and 2013, and were aged 16 or older at the time of diagnosis. AML, NOS; refered to acute myeloid leukemia with FAB or WHO type, not otherwise specified, included acute non-lymphocytic leukemia, acute granulocytic leukemia, acute myelogenous leukemia and acute myelocytic leukemia according to ICD-O-3. b) Patients who had been histologically confirmed, diagnosed and were actively followed up. However, the patients were excluded if they were younger than 16 years; had insufficient or unknown clinicopathologic-profile; had unknown marital status, cause of death or survival information. Finally, 50825 patients were included for analysis in our study.

### Study variables

The following variables were extracted from the SEER database, including marital status, sex, race, age at diagnosis, AML subtype, cause-specific survival (CSS), and adjuvant therapy. Marital status at diagnosis referred to “the status at diagnosis” when not otherwise specified (NOS), and was categorized as married, divorced, widowed, separated and single (never married), and also categorized as a binary variable into married and unmarried (including single, divorced or separated, and widowed groups) in matched case-control analysis. Race was classified into African American, non-Hispanic white, and others (American Indian/AK Native, Asian/Pacific Islander) as provided by the SEER database. Age at diagnosis was divided into different groups: 16 to 35 year, 36 to 55 year, 56 to 75 year, 76 to 95 year, and 96 year or over. Data of AML subtype were all coded according to ICD-O-3. Age at the diagnosis and AML subtype were categorized as a binary variable into 16 to 55 year *vs.* 56 year or over and AML, NOS *vs.* others in matched case-control analysis. Adjuvant therapy was categorized as none radiotherapy, beam radiation or radioisotopes, and radiotherapy unknown.

### Outcome measurement

Previous studies have reported about overall survival (OS), while cancer-caused specific survival (CSS) was neglected. CSS usually provides more accurate information than OS about the death caused by primary cancer. Thus, we in our study only focussed on CSS as the primary outcome, and it was defined from the date of diagnosis to the date of cancer-specific death and was shown as “SEER cause-specific survival”. Deaths attributed to AML were treated as events. Patients who died from other causes or were still alive at the time of the last follow-up were treated as censored observations.

### Statistical analysis

Clinicopathological baseline characteristics were compared with Pearson chi-square test for categorical data. CSS rate was calculated by Kaplan-Meier curve, and compared by log-rank (Mantel-Cox) test. Univariate and multivariate Cox proportional hazard models were built to determine survival outcome and risk factors. To minimize the group differences on covariates between patients who were married, unmarried or never married, a 1:1 propensity score matching was used. Propensity scores were calculated with logistic regression, with multiple imputation and backward elimination with a significance level of 0.05. Matching on the propensity scores was done with a nearest-neighbor algorithm, allowing a maximum tolerated difference between propensity scores of no larger than 0.1 of the propensity score standard deviation [18]. Group differences were estimated by cross table chi-square test. The log-rank test (Kaplan-Meier curve) was applied to estimate median cancer-CSS between matched groups. For this study, data points about sex, race, AML subtype, age at diagnosis and adjuvant therapy were included in propensity matching. The respective matching ratios of 1:1 were selected to maximize the number of matched pairs without exceeding the maximum tolerated difference between matched propensity scores. All statistical analyses were performed using the Statistical Package for the Social Sciences (SPSS) software version 22 (SPSS Inc., Chicago, IL, USA). The *P* value of < 0.05 represented statistically significant difference.

## RESULTS

### Demographic and clinicopathological baseline characteristics

Based on the inclusion criteria, a total of 50,825 eligible patients were identified, including 27,510 male and 23,315 female patients. Among these, 30,006 were married, 8,515 were widowed, 7,927 never married, 3,936 divorced and 441 were separated. Significant differences in demographic and clinicopathological characteristics, including sex, race, age at diagnosis, AML subtype, adjuvant therapy and cause of death were observed in patients from marital status groups. Especially, married and never married patients were more likely to be males compared in other groups. Also the married patients had a better chance to be in age groups of 36-55, 56-75, and 76-95, while patients in younger age group of 16-35, were predominantly never married. Most of the patients were whites. In addition, patients of all marital status groups were mostly diagnosed for AML, NOS and experienced no radiotherapy, and died due to AML. The demographic and clinicopathological characteristics of AML patients with different marital status have been summarized in Table 1.

**Table 1:** Characteristics of AML patients based on marital status (*n*=50825) ^a^

Characteristic	All patients no. (%)	Marital status					
		Married no. (%)	Widowed no. (%)	Never married no. (%)	Divorced no. (%)	Separated no. (%)	*P*-value ^b^
No. of patients	50825(100)	30006(100)	8515(100)	7927(100)	3936(100)	441(100)	-
Sex Male Female	27510(54.1) 23315(45.9)	18990(63.3) 11016(36.7)	2238(26.3) 6267(73.7)	4248(53.6) 3679(46.4)	1809(46.0) 2127(54.0)	215(48.8) 226(51.2)	<0.001
Age 16-35 36-55 56-75 76-95 >95	4540(8.9) 9399(18.5) 20516(40.4) 16176(31.8) 194(0.4)	1529(5.1) 6182(20.6) 13915(46.4) 8354(27.8) 26(0.1)	10(0.1) 153(1.8) 2303(27.0) 5903(69.3) 146(1.8)	2812(35.5) 1916(24.2) 2131(26.9) 1047(13.2) 21(0.2)	137(3.5) 1010(25.7) 1995(50.7) 793(20.1) 1(0.0)	52(11.8) 138(31.3) 172(39.0) 79(17.9) 0(0.0)	<0.001
Race Black White Other (American Indian/AK Native, Asian/Pacific Islander) Unknown	4102(8.1) 42610(83.8) 4013(7.9) 100(0.2)	1646(5.5) 25746(85.8) 2567(8.6) 47(0.1)	655(7.7) 7323(86.0) 528(6.2) 9(0.1)	1261(15.9) 5927(74.8) 703(8.9) 36(0.4)	466(11.8) 3281(83.4) 182(4.6) 7(0.2)	74(16.8) 333(75.5) 33(7.5) 1(0.2)	<0.001
AML subtype AML, NOS Acute promyelocytic leukemia Acute myelomonocytic leukemia AML with RPN1/EVI1 fusion gene AML with CBFB/MYH11 fusion gene AML with RUNX1/RUNX1T1 fusion gene AML with MLLT3/MLL fusion gene AML with RBM15/MKL1 fusion gene AML with DEK/NUP214 fusion gene AML with minimal differentiation AML without maturation AML with maturation AML with myelodysplasia-related changes Acute erythroid leukemia Acute megakaryoblastic leukemia Therapy-related myeloid neoplasm	30005(59.0) 3833(7.5) 4942(9.7) 22(0.0) 503(1.0) 619(1.2) 280(0.6) 21(0.0) 28(0.0) 1203(2.4) 1922(3.8) 2243(4.4) 3132(6.2) 864(1.7) 312(0.6) 896(1.9)	17442(58.1) 2284(7.6) 2958(9.9) 13(0.0) 283(0.9) 353(1.2) 141(0.5) 14(0.0) 15(0.0) 703(2.3) 1161(3.9) 1325(4.4) 2009(6.9) 546(1.8) 190(0.6) 569(1.9)	5702(67.0) 287(3.4) 801(9.4) 4(0.0) 30(0.4) 54(0.6) 44(0.5) 2(0.0) 3(0.0) 224(2.6) 274(3.2) 322(3.8) 515(6.0) 122(1.4) 38(0.4) 95(1.3)	4317(54.5) 951(12.0) 757(9.5) 3(0.0) 126(1.6) 142(1.8) 65(0.8) 3(0.0) 10(0.0) 182(2.3) 329(4.2) 373(4.7) 356(4.5) 112(1.4) 59(0.7) 142(2.0)	2301(58.5) 271(6.9) 378(9.6) 0(0) 56(1.4) 61(1.5) 27(0.7) 2(0.0) 0(0.0) 87(2.2) 146(3.7) 196(5.0) 228(5.8) 78(2.0) 22(0.6) 79(2.1)	243(55.1) 40(9.1) 48(10.9) 2(0.6) 8(1.8) 9(1.8) 3(0.7) 0(0.0) 0(0.0) 7(1.6) 12(2.7) 27(6.1) 24(6.1) 6(1.4) 3(0.7) 11(2.4)	<0.001
Adjuvant therapy None Beam radiation or radioisotopes Unknown	48683(95.8) 1896(3.7) 246(0.5)	2867(95.6) 1201(4.0) 134(0.4)	8390(98.5) 64(0.8) 61(0.7)	7430(93.7) 467(5.9) 30(0.4)	3772(95.8) 146(3.7) 18(0.5)	420(95.2) 18(4.1) 3(0.7)	<0.001
Cause of Death Alive or dead of other cause Dead (attributable to AML) Not first tumor	11317(22.3) 27406(53.9) 12102(23.8)	6653(22.2) 15891(53.0) 7462(24.8)	895(10.5) 5285(62.1) 2335(27.4)	2819(35.6) 3829(48.3) 1279(16.1)	829(21.1) 2170(55.1) 937(23.8)	121(27.4) 231(52.4) 89(20.2)	<0.001

Abbreviation: NOS, no other specific; SEER, Surveillance, Epidemiology and End Results; AML, acute myeloid leukemia.

^a^Data represented number of patients.

^b^*P* value of the Chi-square test or Wilcoxon-Mann-Whitney test refers to comparison for the differences in proportions among subgroups.

### Effect of marital status and other variables on cancer-caused specific survival

**Figure 1:**
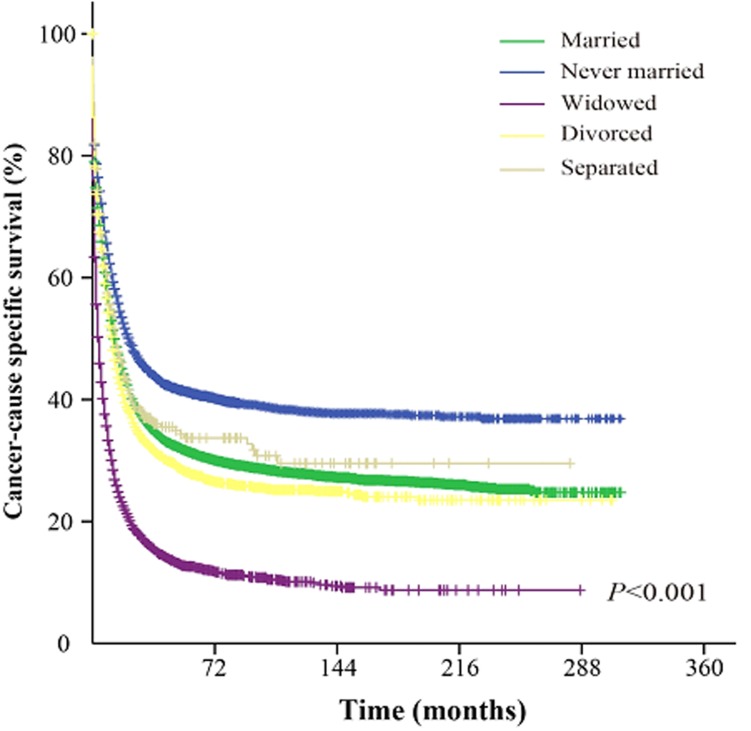
Survival curves of AML patients based on their marital status χ^2^ = 2097.9.

The univariate analysis showed that never married group AML patients had better cancer-CSS than married, widowed, divorced and separated patients. The median cancer-CSS in this group was 22 months, while it was 13 months in married group, 14 months in separated group, 4 months in widowed group and 12 months in divorced group patients. This difference was statistically significant according to the univariate log-rank test (*P* < 0.001) (Figure. 1). In addition, among the demographic and clinicopathological variables, sex, age, race, AML subtype, adjuvant therapy, and marital status were identified as independent factors for predicting CSS based on univariate analysis (Table 2). However, multivariate analysis with Cox regression model indicated sex (male, HR 1.085, 95%CI: 1.058-1.111, *P* < 0.001), age ( > 55 years, HR 2.352, 95%CI: 2.283-2.423, *P* < 0.001), AML subtype (others, HR 0.727, 95%CI: 0.709-0.745, *P* < 0.001), adjuvant therapy (radiation, HR 0.751, 95%CI: 0.704-0.800, *P* < 0.001), and marital status (married, HR 0.802, 95%CI: 0.782-0.822, *P* < 0.001) as independent prognostic factors. In addition, further multivariate analysis based on subgroups like age, AML subtype and marital status, again validated these independent prognostic factors. For example, age, 36-55 years (HR 1.426, 95%CI: 1.342-1.508, *P* < 0.001); age, 56-75 years (HR 2.331, 95%CI: 2.205-2.464, *P* < 0.001); age, 76-95 years (HR 3.899, 95%CI: 3.677-4.134, *P* < 0.001); age, > 95 years (HR 5.603, 95%CI: 4.665-6.730, *P* < 0.001); diagnosed as acute promyelocytic leukemia (HR 0.375, 95%CI: 0.352-0.399, *P* < 0.001); diagnosed as acute myelomonocytic leukemia (HR 1.056, 95%CI: 1.015-1.097, *P* = 0.006); diagnosed as AML with CBFB/MYH11 fusion gene (HR 0.465, 95%CI: 0.398-0.542, *P* < 0.001); diagnosed as AML with RUNX1/RUNX1T1 fusion gene (HR 0.607, 95%CI: 0.536-0.687, *P* < 0.001); diagnosed as AML with MLLT3/MLL fusion gene (HR 0.820, 95%CI: 0.690-0.975, *P* = 0.025); diagnosed as AML without maturation (HR 0.913, 95%CI: 0.859-0.970, *P* = 0.003); diagnosed as AML with maturation (HR 0.854, 95%CI: 0.807-0.903, *P* < 0.001); diagnosed as AML with myelodysplasia-related changes (HR 0.716, 95%CI: 0.679-0.756, *P* < 0.001); diagnosed as acute erythroid leukemia (HR 0.901, 95%CI: 0.822-0.987, *P* = 0.026); diagnosed as acute megakaryoblastic leukemia (HR 1.216, 95%CI: 1.055-1.402, *P* = 0.007); diagnosed as therapy-related myeloid neoplasm (HR 0.055 95%CI: 0.038-0.078, *P* = 0.007); and marital status (widowed, HR 1.312, 95%CI: 1.267-1.357, *P* < 0.001; never married, HR 1.167, 95%CI: 1.125-1.21, *P* < 0.001; divorced, HR 1.148, 95%CI: 1.098-1.201, *P* < 0.001 and separated, HR 1.145, 95%CI: 1.006-1.304, *P* = 0.041).

**Table 2:** Univariate and multivariate survival analysis of AML patients from SEER database

Variable	Univariate analysis		Multivariate analysis	
	HR (95% CI)	*P*	HR (95% CI)	*P*
Sex (Male *vs.* Female)	1.068 (1.043-1.094)	<0.001	1.085 (1.058-1.111)	<0.001
Age (> 55 *vs.* 16-55 years)	2.428 (2.359-2.500)	<0.001	2.352 (2.283-2.423)	<0.001
Race (Non-White *vs.* White)	0.912 (0.883-0.942)	<0.001	0.987 (0.955-1.020)	0.426
AML subtype (Others *vs.* AML, NOS)	0.674 (0.658-0.691)	<0.001	0.727 (0.709-0.745)	<0.001
Adjuvant therapy (Radiation *vs.* None )	0.578 (0.543-0.616)	<0.001	0.751 (0.704-0.800)	<0.001
Marital Status (Married *vs.* Unmarried )	0.875 (0.855-0.897)	<0.001	0.802 (0.782-0.822)	<0.001
Age (years) 16-35 36-55 56-75 76-95 >95	Reference 1.426 (1.348-1.507) 1.592 (1.552-1.634) 1.648 (1.618-1.679) 1.634 (1.557-1.715)	<0.001 <0.001 <0.001 <0.001	Reference 1.426 (1.342-1.508) 2.331 (2.205-2.464) 3.899 (3.677-4.134) 5.603 (4.665-6.730)	<0.001 <0.001 <0.001 <0.001
Race Black White Other (American Indian/AK Native, Asian/Pacific Islander) Unknown	0.924 (0.884-0.966) Reference 0.952 (0.932-0.974) 0.855 (0.770-0.950)	<0.001 <0.001 0.003	1.008 (0.963-1.054) Reference 1.024 (0.980-1.070) 0.831 (0.607-1.138)	0.740 0.297 0.249
AML subtype AML, NOS Acute promyelocytic leukemia Acute myelomonocytic leukemia AML with RPN1/EVI1 fusion gene AML with CBFB/MYH11 fusion gene AML with RUNX1/RUNX1T1 fusion gene AML with MLLT3/MLL fusion gene AML with RBM15/MKL1 fusion gene AML with DEK/NUP214 fusion gene AML with minimal differentiation AML without maturation AML with maturation AML with myelodysplasia-related changes Acute erythroid leukemia Acute megakaryoblastic leukemia Therapy-related myeloid neoplasm	Reference 0.295 (0.277-0.314) 0.984 (0.965-1.003) 0.887 (0.713-1.102) 0.773 (0.744-0.804) 0.867 (0.846-0.889) 0.942 (0.915-0.970) 0.910 (0.819-1.012) 0.982 (0.946-1.020) 1.006 (0.997-1.015) 0.978 (0.971-0.984) 0.977 (0.972-0.983) 0.978 (0.973-0.983) 0.993 (0.986-1.001) 1.009 (0.998-1.020) 0.810 (0.789-0.830)	<0.001 0.094 0.279 <0.001 <0.001 <0.001 0.082 0.349 0.188 <0.001 <0.001 <0.001 0.087 0.095 <0.001	Reference 0.375 (0.352-0.399) 1.056 (1.015-1.097) 0.795 (0.413-1.527) 0.465 (0.398-0.542) 0.607 (0.536-0.687) 0.820 (0.690-0.975) 0.581 (0.277-1.220) 0.863 (0.490-1.519) 1.070 (0.995-1.152) 0.913 (0.859-0.970) 0.854 (0.807-0.903) 0.716 (0.679-0.756) 0.901 (0.822-0.987) 1.216 (1.055-1.402) 0.055 (0.038-0.078)	<0.001 0.006 0.490 <0.001 <0.001 0.025 0.151 0.609 0.070 0.003 <0.001 <0.001 0.026 0.007 <0.001
Adjuvant therapy None Beam radiation or radioisotopes Unknown	Reference 0.524 (0.490-0.562) 1.136 (1.050-1.229)	<0.001 0.002	Reference 0.703 (0.655-0.754) 1.114 (0.952-1.304)	<0.001 0.179
Marital Status Married Widowed Never married Divorced Separated	Reference 1.813 (1.757-1.871) 0.786 (0.758-0.814) 1.072 (1.025-1.122) 0.942 (0.827-1.073)	<0.001 <0.001 0.002 0.268	Reference 1.312 (1.267-1.357) 1.167 (1.125-1.211) 1.148 (1.098-1.201) 1.145 (1.006-1.304)	<0.001 <0.001 <0.001 0.041

Abbreviation: CSS, cancer-specific death; HR, hazard ratio; CI, confidence interval; AML, acute myeloid leukemia; NOS, no other specific; SEER, Surveillance, Epidemiology and End Results

### Subgroup analysis of the marital status effect on cancer-CSS based on sex, age, AML subtype and adjuvant therapy

Our study has identified multiple variables including, sex, age, AML subtype and adjuvant therapy as risk factors for AML mortality, based on Cox proportional hazard regression model analysis. This observation has also been verified independently by other studies [19-21]. Thus, subsequently we stratified patients from different marital status into subgroups based on these variables and studied their effect on cancer-CSS. Our analysis revealed that patients from widowed and never married groups correlated with poor CSS, in comparison to married group, after all these patients were adjusted for the aforementioned variables (*P* < 0.05). In addition, divorced group patients also displayed increased risk for cancer-caused specific mortality, in comparison to married group patients, after adjustment for all variables except in radiation subgroup (HR 1.134, 95% CI: 0.898-1.430, *P* = 0.291). However, the difference between married and separated group patients was not apparent in the majority of the subgroups (Table 3).

**Table 3:** Univariate and multivariate CSS analysis, based on sex, age, AML subtype and adjuvant therapy, of AML patients with different marital status

Variable	Univariate analysis		Multivariate analysis	
	HR (95% CI)	*P*	HR (95% CI)	*P*
Sex Male Married Widowed Never married Divorced Separated Female Married Widowed Never married Divorced Separated	Reference 1.623 (1.533-1.719) 0.763 (0.729-0.800) 1.098 (1.031-1.170) 0.976 (0.814-1.170) Reference 2.115 (2.029-2.205) 0.846 (0.801-0.894) 1.131 (1.061-1.207) 0.976 (0.810-1.176)	<0.001 <0.001 0.004 0.790 <0.001 <0.001 <0.001 0.797	Reference 1.197 (1.129-1.296) 1.157 (1.101-1.215) 1.179 (1.106-1.256) 1.164 (0.971-1.396) Reference 1.318 (1.260-1.379) 1.165 (1.102-1.232) 1.121 (1.051-1.195) 1.131 (0.939-1.363)	<0.001 <0.001 <0.001 0.101 <0.001 <0.001 0.001 0.196
Age (years) 16-55 Married Widowed Never married Divorced Separated >55 Married Widowed Never married Divorced Separated	Reference 1.320 (1.066-1.635) 0.981 (0.928-1.037) 1.273 (1.166-1.390) 1.116 (0.903-1.380) Reference 1.505 (1.457-1.555) 1.111 (1.058-1.167) 1.043 (0.990-1.099) 1.071 (0.098-1.262)	0.011 0.501 <0.001 0.308 <0.001 <0.001 0.115 0.417	Reference 1.320 (1.065-1.636) 1.146 (1.079-1.218) 1.265 (1.158-1.382) 1.176 (0.952-1.453) Reference 1.279 (1.233-1.326) 1.139 (1.085-1.197) 1.116 (1.059-1.176) 1.123 (0.952-1.324)	0.011 <0.001 <0.001 0.134 <0.001 <0.001 <0.001 0.169
AML subtype AML, NOS Married Widowed Never married Divorced Separated Others Married Widowed Never married Divorced Separated	Reference 1.745 (1.679-1.813) 0.811 (0.775-0.849) 1.078 (1.019-1.141) 1.038 (0.881-1.223) Reference 1.827 (1.728-1.932) 0.771 (0.728-0.816) 1.064 (0.989-1.146) 0.852 (0.689-1.054)	<0.001 <0.001 0.009 0.654 <0.001 <0.001 0.098 0.141	Reference 1.321 (1.267-1.377) 1.157 (1.104-1.213) 1.154 (1.090-1.222) 1.255 (1.065-1.478) Reference 1.292 (1.216-1.372) 1.180 (1.112-1.253) 1.135 (1.054-1.222) 1.040 (0.840-1.287)	<0.001 <0.001 <0.001 0.007 <0.001 <0.001 0.001 0.719
Adjuvant therapy None Married Widowed Never married Divorced Separated Beam radiation or radioisotopes Married Widowed Never married Divorced Separated	Reference 1.763 (1.708-1.820) 0.788 (0.760-0.817) 1.066 (1.019-1.116) 0.929 (0.813-1.061) Reference 3.049 (2.430-3.827) 0.874 (0.748-1.020) 1.170 (0.928-1.475) 1.289 (0.728-2.283)	<0.001 <0.001 0.006 0.276 <0.001 0.088 0.185 0.384	Reference 1.299 (1.255-1.345) 1.157 (1.114-1.201) 1.148 (1.097-1.203) 1.119 (0.979-1.278) Reference 1.483 (1.148-1.916) 1.252 (1.052-1.489) 1.134 (0.898-1.430) 1.865 (1.049-3.318)	<0.001 <0.001 <0.001 0.100 0.003 0.011 0.291 0.034

Abbreviation: CSS, cancer-specific death; HR, hazard ratio; CI, confidence interval; AML, acute myeloid leukemia; NOS, no other specific;

### Analysis of cancer-caused specific survival between matched groups

Based on the demographic and clinicopathological variables, propensity scores for unmarried, never married, widowed and divorced patient groups were estimated. Next, the patients were independently matched based on their propensity scores with the married group patients. For instance, the 1:1 matching between unmarried and married group patients resulted in 18,345 matched pairs and a sample size of 36,691 patients. Similarly, the matching between never married and married patients resulted in, 7,697 matched pairs. All the group differences in both these matched datasets did not reach the statistically significance (*P* > 0.05), thereby, representing negligible differences across AML subtypes and all other demographic and treatment variables (Table 4, 5). However, in case of matching of patients from widowed group with married patients, race and adjuvant therapy were categorized as a binary variables to minimize the group difference, and this led to 8,495 matched pairs without group differences except race variable. Finally, the survival analysis was also performed for this matched dataset (Table 6). Importantly, the matching of divorced group with married group could not be done due to the absence of matched dataset across most variables (Appendix Tables 1), and thus no further survival analyses were performed.

**Table 4:** Baseline characteristics of unmarried and married AML patients, before and after propensity matching

Variable	Unmatched (complete) dataset		χ^2^	*P*	Matched (1:1) dataset		χ^2^	*P*
	Unmarried (n=20819)	married (n=30006)			Unmarried (n=18346)	married (n=18345)		
Age (years) 16-55 > 55	6228(29.9%) 14591(70.1%)	7711(25.7%) 22295(74.3%)	109.803	<0.001	5787(31.5%) 12559(68.5%)	5815(31.7%) 12530(86.3%)	0.101	0.751
Race Black White Other Unknown	2456(11.8%) 16864(81.0%) 1446(6.9%) 53(0.3%)	1646(5.5%) 25746(85.8%) 2567(8.6%) 47(0.1%)	686.708	<0.001	1464(8.0%) 15440(84.2%) 1411(7.7%) 31(0.1%)	1431(7.8%) 15442(84.2%) 1442(7.9%) 30(0.1%)	0.729	0.866
Sex Male Female	8520(40.9%) 12299(59.1%)	18990(63.3%) 11016(36.7%)	2475.636	<0.001	8380(45.7%) 9966(54.3%)	8379(45.7%) 9966(54.3%)	0.000	1.000
AML subtype AML, NOS other	12563(60.3%) 8256(39.7%)	17442(58.1%) 12564(41.9%)	24.948	<0.001	10705(58.4%) 7641(41.6%)	10675(58.2%) 7670(41.8%)	0.097	0.755
Adjuvant therapy None Beam radiation or radioisotopes Unknown	20012(96.1%) 695(3.3%) 112(0.6%)	28671(95.6%) 1201(4.0%) 134(0.4%)	17..079	<0.001	17603(96.0%) 662(3.6%) 81(0.4%)	17572(95.8%) 693(3.8%) 80(0.4%)	0.743	0.690

Abbreviation: AML, acute myeloid leukemia; NOS, no other specific.

**Table 5:** Baseline characteristics of never married and married AML patients, before and after propensity matching

Variable	Unmatched (complete) dataset		χ^2^	*P*	Matched (1:1) dataset		χ^2^	*P*
	never married (n=7927)	married (n=30006)			never married (n=7697)	married (n=7697)		
Age (years) 16-55 > 55	4728(59.6%) 3199(40.4%)	7711(25.7%) 22295(74.3%)	3278.608	<0.001	4502(58.5%) 3195(41.5%)	4502(58.5%) 3195(41.5%)	0.000	1.000
Race Black White Other Unknown	1261(15.9%) 5927(74.8%) 703(8.9%) 36(0.4%)	1646(5.5%) 25746(85.8%) 2567(8.6%) 47(0.1%)	1006.249	<0.001	1043(13.6%) 5926(77.0%) 703(9.1%) 25(0.3%)	1043(13.6%) 5905(76.7%) 724(9.4%) 25(0.3%)	0.346	0.951
Sex Male Female	4248(53.6%) 3679(46.4%)	18990(63.3%) 11016(36.7%)	248.520	<0.001	3509(45.6%) 4188(54.4%)	3488(45.3%) 4209(54.7%)	0.116	0.734
AML subtype AML, NOS other	4317(54.5%) 3610(45.5%)	17442(58.1%) 12564(41.9%)	34.511	<0.001	4192(54.5%) 3505(45.5%)	4192(54.5%) 3505(45.5%)	0.000	1.000
Adjuvant therapy None Beam radiation or radioisotopes Unknown	7430(93.7%) 467(5.9%) 30(0.4%)	28671(95.6%) 1201(4.0%) 134(0.4%)	53..727	<0.001	7216(93.8%) 455(5.9%) 26(0.3%)	7200(93.5%) 466(6.1%) 31(0.4%)	0.588	0.745

Abbreviation: AML, acute myeloid leukemia; NOS, no other specific.

**Table 6:** Baseline characteristics of widowed and married AML patients, before and after propensity matching

Variable	Unmatched (complete) dataset		χ^2^	*P*	Matched (1:1) dataset		χ^2^	*P*
	widowed (n=8515)	married (n=30006)			widowed (n=8495)	married (n=8495)		
Age (years) 16-55 > 55	163(1.9%) 8352(98.1%)	7711(25.7%) 22295(74.3%)	2307.140	<0.001	163(1.9%) 8332(98.1%)	163(1.9%) 8332(98.1%)	0.000	1.000
Race Non white White	1192(14.0%) 7323(86.0%)	4260(14.2%) 25746(85.8%)	0.215	0.643	1172(13.8%) 7323(86.2%)	1068(12.6%) 7427(87.4%)	5.562	0.018
Sex Male Female	2248(26.3%) 6267(73.7%)	18990(63.3%) 11016(36.7%)	3648.390	<0.001	2248(36.0%) 6247(64.0%)	2248(36.0%) 6247(64.0%)	0.000	1.000
AML subtype AML, NOS other	5702(67.0%) 2813(33.0%)	17442(58.1%) 12564(41.9%)	215.910	<0.001	5682(66.9%) 2813(33.1%)	5714(67.3%) 2781(32.7%)	0.273	0.601
Adjuvant therapy None radiation Beam radiation or radioisotopes	8390(98.5%) 125(1.5%)	28671(95.6%) 1335(4.4%)	161.651	<0.001	8370(98.5%) 125(1.5%)	8372(98.6%) 123(1.4%)	0.016	0.898

Abbreviation: AML, acute myeloid leukemia; NOS, no other specific.

**Figure 2:**
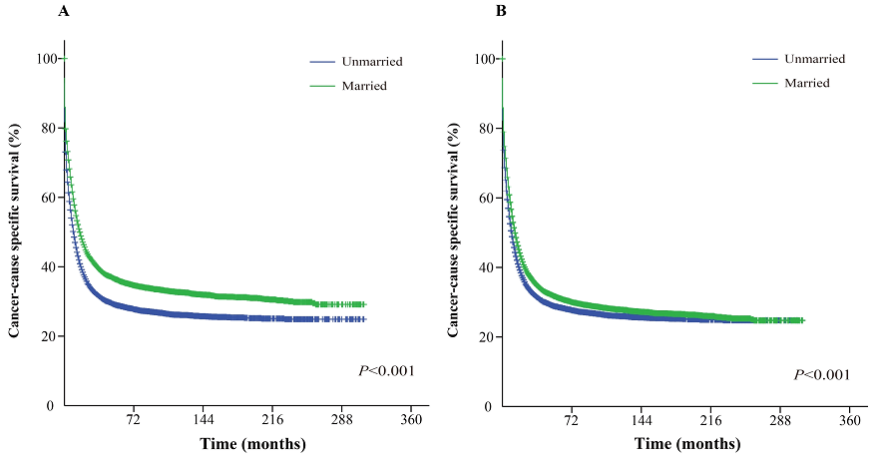
Kaplan-Meier survival curves: The cancer-caused specific survival of unmarried and married groups of matched and unmatched AML patients **A**. matched group, χ^2^ = 295.5; **B.** unmatched group, χ^2^ = 128.1.

**Figure 3:**
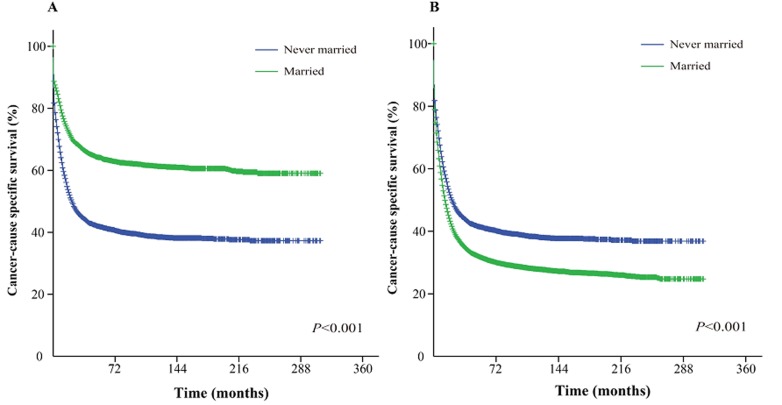
Kaplan-Meier survival curves: The cancer-caused specific survival of never married and married groups of matched and unmatched AML patients **A**. matched group, χ^2^ = 639.6; **B.** unmatched group, χ^2^ = 191.4.

**Figure 4:**
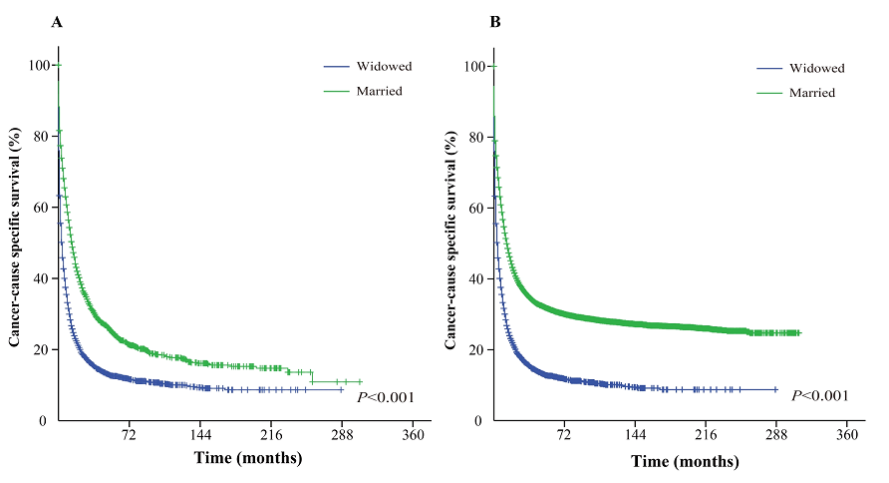
Kaplan-Meier survival curves: The cancer-caused specific survival of widowed and married groups of matched and unmatched AML patients **A**. matched group, χ^2^ = 891.4; **B.** unmatched group, χ^2^ = 1565.2.

The Kaplan-Meier curve based estimation of cancer-caused specific survival for the unmatched and matched marital status groups, was performed as shown in Figure 2, 3 & 4. The survival analysis between unmatched unmarried and married group patients showed a median CSS of 10 months (95% CI: 9.6-10.4) in the unmarried group, while 13 months (95% CI: 12.5-13.5) in the married group patients (*P* < 0.001). A similar analysis between matched unmarried and married groups showed, a median CSS of 10 months (95% CI: 9.5-10.5) and 16 months (95% CI: 15.2-16.8) (*P* < 0.001, Figure. 2) respectively. In addition, the 5-year cancer-caused specific survival between unmarried and married patients was 28.4% and 31.1% in unmatched group, while 28.6% *versus* 35.7% in matched, group, respectively (*P* < 0.001). The never married *versus* married analysis between unmatched groups showed median CSS of 22 months (95% CI: 20.1-23.9) and 13 months (95% CI: 12.5-13.5) respectively (*P* < 0.001). On the contrary, in the matched group, the median CSS was 22 months (95% CI: 20.0-24.0) for never married patients, while the married patients did not achieve the required 50% survival value (*P* < 0.001, Figure. 3). The 5-year cancer-caused specific survival was 40.9% *versus* 31.1% in unmatched and 41.4% *versus* not reached in matched never married and married group patients (*P* < 0.001). In addition, the cancer-CSS analysis between widowed and married patients displayed a median CSS of 4 months (95% CI: 3.7-4.3) and 13 months (95% CI: 12.5-13.5), respectively, and 5-year cancer-caused specific survival of 12.6% and 31.1% respectively in unmatched groups (*P* < 0.001). The similar trends were observed in the matched groups, where the median CSS was 4 months (95% CI 3.7-4.3) *versus* 14 months (95% CI: 13.2-14.8) in widowed *versus* married group patients (*P* < 0.001, Figure. 4). The 5-year cancer-caused specific survival was 12.5% *versus* 23.6%, respectively (*P* < 0.001).

Furthermore, we also evaluated the correlation between different factors and cancer-caused specific survival, using multivariate proportional hazard model by comparing matched datasets to unmatched complete datasets, as summarized in Table 7, 8. Various features were observed in all pairs of matched groups, and among them several independent prognostic factors emerged between matched unmarried *versus* married groups, including age, AML subtype and adjuvant therapy, and were similar to those in unmatched group. However, race (black, HR 0.946, 95% CI: 0.898-0.998, *P* = 0.041; other, HR 0.920, 95% CI: 0.872-0.971, *P* = 0.002; unknown, HR 0.531, 95% CI: 0.335-0.844, *P* = 0.007) was found to be associated with CSS in matched groups but not in unmatched. Similarly, sex was as a prognostic factor in unmatched groups but not in matched groups (*P* > 0.05). Among the never married *versus* married groups analysis, age ( > 55 years, HR 1.611, 95% CI: 1.529-1.698, *P* < 0.001), race (black, HR 1.549, 95% CI: 1.449-1.656, *P* < 0.001), sex (male, HR 1.117, 95% CI: 1.062-1.176, *P* < 0.001) and adjuvant therapy (radiation, HR 0.844, 95% CI: 0.755-0.944, *P* = 0.003) were observed to be associated with CSS in matched groups, while in unmatched groups, some additional prognostic factors were also identified; age ( > 55 years, HR 2.165, 95% CI: 2.094-2.237, *P* < 0.001), race (other, HR 1.053, 95% CI: 1.002-1.106, *P* = 0.041), sex (male, HR 1.160, 95% CI: 1.126-1.194, *P* < 0.001), AML subtype (others, HR 0.733, 95% CI: 0.712-0.754, *P* < 0.001) and adjuvant therapy (radiation, HR 0.716, 95% CI: 0.664-0.771, *P* < 0.001). The age > 55 years and diagnosis of AML, NOS eventually increased the risk of CSS in widowed and married group patients. Being male (HR 1.160, 95% CI: 1.126-1.194, *P* < 0.001) was associated with poor CSS in matched widowed *versus* married groups, while not in unmatched groups (HR 0.998, 95% CI: 0.971-1.025, *P* = 0.887). Also the similar result was observed in adjuvant therapy as receiving radiation (HR 0.725, 95% CI: 0.672-0.783, *P* < 0.001), which appeared as a favorable factor in unmatched group but did not reach statistical significance in matched group (HR 0.871, 95% CI: 0.737-1.030, *P* = 0.106).

**Table 7:** Hazard ratios of CSS predictors in AML patients with different marital status, based on multivariate analysis (matched and unmatched complete datasets)

Variable	Unmarried *vs.* married matched		Unmarried *vs.* married unmatched		Never married *vs.* married matched		Never married *vs.* married unmatched	
	HR (95% CI)	*P*	HR (95% CI)	*P*	HR (95% CI)	*P*	HR (95% CI)	*P*
Age (> 55 *vs.* 16-55 years)	2.438 (2.358-2.522)	<0.001	2.290 (2.223-2.359)	<0.001	1.611 (1.529-1.698)	<0.001	2.165 (2.094-2.237)	<0.001
Race Black White Other Unknown	0.946 (0.898-0.998) Reference 0.920 (0.872-0.971) 0.531 (0.335-0.844)	0.041 0.002 0.007	1.003 (0.959-1.048) Reference 1.013 (0.969-1.058) 0.776 (0.567-1.062)	0.912 0.574 0.113	1.549 (1.449-1.656) Reference 1.018 (0.931-1.114) 0.778 (0.461-1.315)	<0.001 0.692 0.349	1.030 (0.976-1.087) Reference 1.053 (1.002-1.106) 0.750 (0.521-1.079)	0.285 0.041 0.121
Sex (Male *vs.* Female)	1.012 (0.984-1.040)	0.420	1.036 (1.012-1.061)	0.004	1.117 (1.062-1.176)	<0.001	1.160 (1.126-1.194)	<0.001
AML subtype (Others *vs.* AML, NOS)	0.741 (0.720-0.763)	<0.001	0.725 (0.708-0.744)	<0.001	0.958 (0.910-1.008)	0.102	0.733 (0.712-0.754)	<0.001
Adjuvant therapy None Radiation Unknown	Reference 0.683 (0.627-0.743) 1.172 (0.966-1.418)	<0.001 0.101	Reference 0.693 (0.646-0.743) 1.215 (1.038-1.422)	<0.001 0.016	Reference 0.844 (0.755-0.944) 1.286 (0.861-1.921)	0.003 0.219	Reference 0.716 (0.664-0.771) 1.162 (0.948-1.425)	<0.001 0.149

Abbreviation: CSS, cancer-specific death; HR, hazard ratio; CI, confidence interval; AML, acute myeloid leukemia; NOS, no other specific.

**Table 8:** Hazard ratios of CSS predictors by multivariate analysis in widowed *vs.* **married AML patients (matched and unmatched)**

Variable	widowed *vs.* married matched		widowed *vs.* married unmatched	
	HR (95% CI)	*P*	HR (95% CI)	*P*
Sex (Male *vs.* Female)	0.570 (0.538-0.604)	<0.001	0.998 (0.971-1.025)	0.887
Age (> 55 *vs.* 16-55 years)	3.593 (2.904-4.445)	<0.001	2.339 (2.252-2.429)	<0.001
Race (Non-White *vs.* White)	0.977 (0.920-1.037)	0.445	0.988 (0.950-1.028)	0.552
AML subtype (Others *vs.* AML, NOS)	0.807 (0.771-0.844)	<0.001	0.733 (0.712-0.754)	<0.001
Adjuvant therapy (Radiation *vs.* None )	0.871 (0.737-1.030)	0.106	0.725 (0.672-0.783)	<0.001

Abbreviation: CSS, cancer-specific death; HR, hazard ratio; CI, confidence interval; AML, acute myeloid leukemia; NOS, no other specific.

## DISCUSSION

In general, our study has been able to show that patients with unmarried status including those who were widowed or divorced have relatively greater risk of death due to primary AML, in comparison to patients who were married. In addition, we also observed that the specific association between marital status and survival outcomes was significant. Consistent with our data, similar conclusions have also been achieved in patients with solid tumors like, lung cancer, colorectal cancer, pancreatic cancer, liver cancer, esophagus cancer, head/neck cancer, non-Hodgkin lymphoma, thyroid cancer, gastric cancer and cervical cancer [7, 9, 11, 14, 22-25]. However, contrary to these observations, the study by Fintel AE [16] demonstrated that marital status did not influence the outcomes of acute lymphoblastic leukemia, while intrinsic differences in disease and disease-specific therapies did. Thus, we have directly compared the published HRs for the overall survival benefit of conventional chemotherapy and/or hematopoietic stem cell transplantation (HSCT) from meta-analysis and systematic reviews, and observed that HRs for the cancer-specific survival benefit correlated with marriage in this study (Table 9). The survival benefit associated with marriage was greater than the several published survival benefits of various treatments (high doses of daunorubicin, high dose of cytarabine, Gemtuzumab ozogamicin included chemotherapy, idarubicin with cytarabine, autologous HSCT and Allogeneic HSCT with reduced-intensity conditioning).

**Table 9:** Comparison of HRs for overall survival associated with conventional treatments (based on prior literature), and with cancer-specific survival associated with marriage (in the present study) in AML patients

Type of Treatment Study	Chemotherapy or Hematopoietic stem cell transplantation	Reference	HR for Treatment (95% CI)	HR for Marriage in Present Study
Meta-Analysis of Prospective Studies [45]	Allogeneic HSCT	Non-allogeneic HSCT (autologous HSCT or chemotherapy	0.76 (0.61-0.95)	0.802 (0.782-0.822)
Meta-Analysis of Prospective Clinical Trials [46]	High doses of daunorubicin	Standard doses of daunorubicin or idarubicin	0.88 (0.79-0.99)	
Systematic Review and Meta-analysis [47]	High dose of cytarabine	Allogeneic HSCT or autologous HSCT	1.66 (1.30-2.14)	
Meta-analysis of Prospective Randomized Phase III Trials [48]	Gemtuzumab ozogamicin included regimens	Non Gemtuzumab ozogamicin included regimens	0.93 (0.86-1.00)	
Systematic Review and Meta-analysis [49]	Conventional chemotherapy with Gemtuzumab ozogamicin	Conventional chemotherapy alone	0.95 (0.83-1.08)	
Meta-analysis of Randomized Clinical Trials [50]	Idarubicin with cytarabine	Daunorubicin with cytarabine	0.88 ( 0.81-0.95)	
Meta-analysis of Randomized Trials [51]	Autologous HSCT	Non- autologous HSCT	1.05 (0.91-1.21)	
Systematic Review and Meta-analysis [52]	Allogeneic HSCT	Intensive or less intensive chemotherapy	0.58 (0.51-0.64)	
Meta-Analysis of Retrospective Studies [53]	Allogeneic HSCT with reduced-intensity conditioning	Allogeneic HSCT with myeloablative conditioning	0.97 (0.88-1.07)	

Abbreviation: HSCT, hematopoietic stem cell transplantation; HR, hazard ratio; CI, confidence interval.

Importantly, we also observed some additional correlations in our study. Like, being never married was observed to be associated with favorable cancer-caused specific survival in general and in almost each subgroup adjusted by each prognostic variable in the univariate and log-rank analysis, when compared with married status. However, the multivariate analysis showed that never married AML patients actually had worse CSS in comparison to married status, and this might be attributed to the fact that population of being never married consisted of relatively more patients aged 15-55 years and were females. These 2 variables were later observed to be independent prognostic factors for survival. Thus, the result was adjusted when independent prognostic factors were integrated in the multivariate analysis. Our hypothesis was further validated when 1:1 matched groups were analyzed similarly using demographic and clinicopathological variables. The married patients showed remarkably better cancer-caused specific survival than never married patients in the log-rank test. The results from matched groups largely simulated the conditions of prospective study in the limited retrospective database, and were more persuasive than unmatched data [18]. Surprisingly, being male did not seem to influence the survival outcome in the matched married *versus* unmarried groups, while it actually increased the risk in unmatched groups. More interestingly, it even protected patients from AML-caused specific death in the matched widowed *versus* married groups. As this database included more males than females, thus it could be likely for males to display a little greater risk (HR 1.068) than females. As AML has not been a malignant tumors with significant gender differences [26], we do not regard sex as a remarkable prognostic factor in AML patients. Also it was noticed that when being widowed, male patients trends to act more optimistically towards disease and emerged with solid economic capability than female patients [4, 5, 9]. Hence, it was no surprise that male patients may survive a while longer in matched widowed *versus* married groups. Notably, it is quite common that unmarried adults usually live “with other persons” in modern society, which SEER database failed to record. So, we suggested that prognostic factors may differ in diverse marital status groups, and living with someone other than a spouse might not confer the similar protective benefit as marriage. Overall, our study emphasized about the substantial impact of marriage or more accurately social support on AML survival. Thus, it can be deduced from our study that providing social support to vulnerable populations such as single or widowed patients, could considerably increase the ratio of positive remission and survival.

Notably, socio-demographic factors have actually been shown to impact the disease outcome in multiple health conditions especially in countries and regions with limited access to free care [27, 28]. Marital status can have positive effect on AML diagnosis and subsequent treatment, as spouses can definitely advice patients to pay more medical attention for suspicious symptoms. Not only this, spouses can also play an important role in management of the disease [29]. Many studies have attempted to explain the reason for the correlation of married status with better survival in cancer by adjusting demographics, stage and treatment, and one reason which seems to be probable is that married patients adhere to the prescribed treatments better than unmarried [30-32]. There are potentially additional underlying etiologies which can explain the benefits of marriage on cancer-cause specific survival. Since the diagnosis of hematological malignancies usually results in more grieved outcome than other hematological diagnoses [33, 34], but it has been observed that married patients showed lower risk of major depression or anxiety than their unmarried counterparts [35], as emotional burden is shared by an intimate partner. Pessimism is another negative mediator between marital status and adherence to therapeutic approaches. Patients with depression generally undergo authoritative treatment less often and thus would display poor survival outcome [36-38]. With the change of marital status, the patient situation appears to become more complicated. It is generally expected that married patients may benefit in terms of emotional and social support in comparison to others who are widow, divorce or separate, as these patients definitely undergo more social and financial stress. As a result these patients cannot cope with stress and develop a negative attitude towards disease or even life, and should not be overlooked. It can be advised that physicians should screen unmarried AML patients, especially those who are experiencing marital upheaval and display pessimistic attitude. In addition they should be referred to mental health specialists, if typical symptoms are identified.

Many studies investigating the impact of marriage usually focus on patients with a single cancer just like the current study. However, there are some additional population-based studies which evaluated the impact of marriage on patient’s outcomes in numerous cancers. The studies by Goodwin *et al.* [39] and Lai *et al.* [40] concluded that marital status has very limited effect on overall survival in cancer patients. The study by Aizer *et al.* [7] proposed a novel view about the significant correlation between marital status and cancer-specific mortality for many cancers including 10 leading malignancies, and their evaluation was based on 1,260,898 contemporary cancer patients throughout the United State. These results were consistent with our study and support our conclusions.

However, there were also few potential limitations of our study. First, our result could not be extended to AML patients from Asia, African, Latin America or even Europe. Second, our study lacked the data related to chemotherapy or HSCT. Third, some patients who cohabitated with a partner privately in the absence of marriage, were regarded as unmarried by SEER database, but these patients might survive longer than actually unmarried patients, hence has the tendency to bias our results. Finally, there was no information about the patients addiction to alcohol, smoke or maybe drug abuse in the SEER database, and these factors can also impact the survival of AML patients [41, 42]. The studies by Park B *et al.* and Balekang GB *et al.* have indicated that patients with unmarried status were more prone to such addiction habits [43, 44]. So, the physician should pay attention to such adverse factors, particularly in unmarried AML patients. Nevertheless, despite these limitations, our study still highlighted the importance of social support as well as marital status, in significantly improving the therapeutic effects in unmarried AML patients.

Overall, our study demonstrated that unmarried AML patients are at a greater risk of cancer-specific mortality, and physicians should definitely evaluate the information about the social status/support of these patients, and when required should counsel and provide health resources targeting towards social support. This intervention may help to improve the rate of cancer-caused specific mortality in unmarried AML patients.

## SUPPLEMENTARY MATERIALS TABLE


