# Supplementary material for: Impact of marital status during diagnosis on cancer-caused specific survival in acute myeloid leukemia patients: a case-control and population-based study
Source: Oncotarget. 2017 Apr 9;8(37):62666–80. doi: 10.18632/oncotarget.16989 (PMC5617539; doi:10.18632/oncotarget.16989)
Supplement: Supplementary file 1 [file oncotarget-08-62666-s001.pdf]

Impact of marital status during diagnosis on cancer-caused specific survival in acute myeloid leukemia patients: a case-control and population-based study

Supplementary Material

Appendix Tables 1: Baseline characteristics for divorced patients versus married patients before and after propensity matching

Variable	Unmatched (complete) dataset		χ^2	*P*	Matched (1:1) dataset		χ^2	*P*
	divorced (n=3936)	married (n=30006)			divorced (n=3936)	married (n=3936)		
Age (years)			21.387	0.001			93.363	0.001
16-55	1147(29.1%)	7711(25.7%)			1147(29.1%)	1554(39.5%)		
> 55	2789(70.9%)	22295(74.3%)			2789(70.9%)	2382(60.5%)		
Race			16.785	0.001			117.896	0.001
Non Caucasus	655(16.6%)	4260(14.2%)			655(16.6%)	1052(26.7%)		
Caucasus	3281(83.4%)	25746(85.8%)			3281(83.4%)	2884(73.3%)		
Sex			440.260	0.001			0.000	1.000
Male	1809(46.0%)	18990(63.3%)			1809(45.6%)	1809(45.6%)		
Female	2127(54.0%)	11016(36.7%)			2127(54.4%)	2127(54.4%)		
AML subtype			0.158	0.691			437.619	0.001
AML, NOS	2301(58.5%)	17442(58.1%)			2301(58.5%)	1375(34.9%)		
other	1635(41.5%)	12564(41.9%)			1635(41.5%)	2561(65.1%)		
Adjuvant therapy			0.658	0.417			425.059	0.001
None radiation	3772(95.8%)	28671(95.6%)			3772(95.8%)	3186(80.9%)		
Beam radiation or radioisotopes	164(4.2%)	1335(4.4%)			164(4.2%)	750(19.1%)		

Abbreviation: AML, acute myeloid leukemia; NOS, no other specific.
